# Renoprotective Effect of Thai Patients with Type 2 Diabetes Mellitus Treated with SGLT-2 Inhibitors versus DPP-4 Inhibitors: A Real-World Observational Study

**DOI:** 10.1155/2023/5581417

**Published:** 2023-05-15

**Authors:** Apichaya Chanawong, Suriyon Uitrakul, Supatcha Incomenoy, Natnicha Poonchuay

**Affiliations:** ^1^Department of Pharmaceutical Care, School of Pharmacy, Walailak University, Tha Sala, Nakhon Si Thammarat 80160, Thailand; ^2^Drug and Cosmetics Excellence Center, Walailak University, Tha Sala, Nakhon Si Thammarat 80161, Thailand

## Abstract

**Background:**

Recently, there is a lack of studies comparing the renoprotective effects of sodium-glucose cotransporter-2 (SGLT-2) inhibitors and dipeptidyl peptidase-4 (DPP-4) inhibitors. This study therefore aimed to investigate the renoprotective effects of SGLT-2 inhibitors and DPP-4 inhibitors on Thai patients with type 2 diabetes mellitus.

**Methods:**

Patient medication records of all patients who used those two antidiabetic classes at Fort Wachirawut Hospital were reviewed. Renal function tests, blood glucose levels, and other baseline characteristics were collected. Continuous variables were compared within the group using the Wilcoxon signed-rank test and between groups using the Mann–Whitney *U* test.

**Results:**

There were 388 and 691 patients with SGLT-2 inhibitors and DPP-4 inhibitors, respectively. The mean estimated glomerular filtration rate (eGFR) of the SGLT-2 inhibitor group was significantly lower from baseline at 18 months of treatment, as well as the DPP-4 inhibitor group. However, the trend of eGFR reduction in patients with baseline eGFR <60 mL/min/1.73 m^2^ was smaller than those with baseline eGFR ≥60 mL/min/1.73 m^2^. In addition, the fasting blood sugar and haemoglobin A1c levels significantly decreased from baseline in both the groups.

**Conclusions:**

Both SGLT-2 inhibitors and DPP-4 inhibitors showed the same trends of eGFR reductions from baseline in Thai patients with type 2 diabetes mellitus. However, SGLT-2 inhibitors should be considered in patients with impaired renal function rather than in all T2DM patients.

## 1. Introduction

Type 2 diabetes mellitus (T2DM) is a major public health problem, with an estimated global prevalence rising to 10.2% (578 million people) by 2030. Poor glycaemic control could mediate endothelial dysfunction, macrovascular and microvascular complications, and increased mortality [1]. To date, the principal goal of T2DM therapy is extended from reducing blood glucose to preventing cardiovascular diseases and related complications. Chronic kidney disease (CKD) is a common comorbidity affecting approximately 50% of patients with T2DM [2, 3]. Patients with diabetic kidney disease should receive comprehensive therapy to lessen the risk of kidney disease progression and cardiovascular diseases [2].

Sodium-glucose cotransporter-2 inhibitors (SGLT2is) are a novel class of glucose-lowering agents that could be used in any stage of T2DM. Beyond improving glycaemic control, several large randomised control trials (RCTs) demonstrated that SGLT2i, i.e., canagliflozin, empagliflozin, and dapagliflozin, had beneficial cardiovascular and renoprotective effects on patients with T2DM. Evidence from those RCTs indicated that SGLT2i therapy was associated with a reduced risk of albuminuria and other renal events with long-term preservation of eGFR [4–6]. According to American Diabetes Association 2022, SGLT2i is now recommended as a first-line agent for diabetes patients with established or high-risk atherosclerotic cardiovascular disease (ASCVD), heart failure, and chronic kidney disease (CKD) and is the prioritised add-on agent to metformin [7]. Recently, eight SGLT2i are available worldwide; however, only four of them are available in Thailand currently, including dapagliflozin, empagliflozin, canagliflozin, and luseogliflozin [8].

Dipeptidyl peptidase-4 inhibitors (DPP4is) are another novel class of glucose-lowering drugs that are recommended as a second-line or add-on to metformin to reduce HbA1c in T2DM patients [7]. It has been reported that DPP4i reduced the risk of albuminuria without other renal benefits compared to placebos [9, 10]. Recently, these two glucose-lowering classes have been more frequently prescribed in routine clinical practice, but only few studies directly compared the effectiveness of these two classes, in particular renoprotective benefits [11–14].

Although some of the studies showed the extent of eGFR changes over time after initiating these drugs, it should be noted that the glucose-lowering effects and adverse effects of SGLT2i and DPP4i vary among different ethnic groups [15, 16]. Even though several large RCTs established the renoprotective benefits of SGLT2i and DPP4i in patients with T2DM, real-world data on these drugs in the Southeast Asian population are still lacking. Studying drug effects in routine clinical settings with a broader population is still considered important as another reference for local patients because, in reality, patients might receive a variety of drugs for their underlying diseases and some drugs might interfere with the renal function of the patients. In addition, the condition that contributes to a decline renal function in real clinical settings is different from clinical trials.

Therefore, the present study aimed to evaluate the renoprotective effects of SGLT2i compared with DPP4i by comparing the changes in eGFR levels and the glucose-lowering effects in the patients receiving either drug in a real clinical setting.

## 2. Materials and Methods

### 2.1. Study Design and Setting

This retrospective cohort study was performed at Fort Wachirawut Hospital, Nakhon Si Thammarat, Thailand. The electronic medication records (EMRs) of all eligible patients were reviewed, and all relevant data were gathered. In Fort Wachirawut Hospital, the available DPP4is were only vildagliptin and linagliptin and the available SGLT2is were only dapagliflozin and empagliflozin. As the SGLT2i drugs have been introduced and used in this hospital since 2017, this study reviewed the EMRs of the patients who started the drugs from 2017 to 2020.

### 2.2. Inclusion and Exclusion Criteria

The present study included patients with type 2 diabetes mellitus who have been prescribed one or more oral glucose-lowering agents, including either a DPP4i or an SGLT2i. Patients who used insulin therapy, used both DPP4i and SGLT2i, had no laboratory data on the first date of DPP4i or SGLT2i prescription, and had only the baseline laboratory data were excluded from the analysis.

### 2.3. Study Variables and Outcomes

The variables that were collected from the patient EMRs included gender, age, weight, height, blood pressure, smoking status, comorbidities, concurrent medications, and laboratory data that are fasting blood sugar (FBS), HbA1c, estimated glomerular filtration rate (eGFR), serum creatinine (SCr), blood urea nitrogen (BUN), LDL cholesterol, HDL cholesterol, triglyceride, total cholesterol, aspartate aminotransferase (AST), and alanine aminotransferase (ALT).

The primary outcome of this study was the changes in posttreatment eGFR compared to the pretreatment of the drugs within the same group at 3, 6, 9, 12, and 18 months of treatment. Additionally, the difference in the eGFR change between the two groups was compared. The magnitude of change was considered using either the actual number of the eGFR levels or the proportion (percentage) of changes in the eGFR level. The “change” and “difference” in eGFR were reported with the dash symbol for a negative change, while the “decrease” and “reduction” were reported without the symbol because it already reflects a negative change.

The secondary outcomes were the changes in eGFR levels among the patients with renal dysfunction; impaired renal function was defined as an eGFR level less than 60 mL/min/1.73 m^2^. The changes in other variables pretreatment and posttreatment of the drugs, including FBS and HbA1c, were also defined as the secondary outcomes.

### 2.4. Statistical Analysis

Baseline characteristics of the patients were described as a percentage, mean, and 95% confidence interval (95% CI) using descriptive statistics. A comparison of primary outcomes between posttreatment and pretreatment within the same group was performed using the Wilcoxon signed-rank test, while a comparison between drug groups was performed using the Mann–Whitney *U* test. The secondary outcomes between drug groups were compared using the Mann–Whitney *U* test. Statistical analyses were performed using SPSS version 28, and statistical significance was set at <0.05.

### 2.5. Ethical Approval

The methodology of the present study was approved by the Human Research Ethics Committee of Walailak University (HREC WU; registration number WUEC-21-168-01). According to the HREC WU regulation, any research that involves only routinely collected information, such as medical charts, does not require patient-informed consent. However, this study's patient data confidentiality and compliance were performed according to the Declaration of Helsinki.

## 3. Results

From January 2017 to December 2020, a total of 504 patients who initiated SGLT2i (279 received dapagliflozin and 225 received empagliflozin) and a total of 931 patients who initiated DPP4i (431 received vildagliptin and 500 received Linagliptin) were screened. Of the total, 388 patients were recruited in the SGLT-2 inhibitor group (dapagliflozin, *n* = 227; empagliflozin, *n* = 161) and 691 patients were recruited in the DPP-4 inhibitor group (vildagliptin, *n* = 321; linagliptin, *n* = 370). The data were analysed as a group of drugs, i.e., SGLT2is (dapagliflozin and empagliflozin) and DPP4is (linagliptin and vildagliptin).

### 3.1. Baseline Characteristics

The ethnicity of the patients in both the groups was documented as Thai. The baseline characteristics of the two groups are presented in Table 1. The average ages, BMI, and blood pressure levels were similar between the SGLT2i and DPP4i groups, as well as the percentages of male patients, comorbidities, and most concurrent medications. However, the differences in baseline characteristics between the two groups were observed in baseline renal function and the proportion of patients who never smoked or had concurrent NSAIDs and aspirin prescriptions. A higher proportion of patients who used NSAIDs and aspirin were reported in the SGLT2i group than in the DPP4i group (55.93% and 42.11%, respectively). In addition, the percentage of nonsmoking patients was higher in the SGLT2i group than in the DPP4i group (75.77% and 65.27%, respectively). The proportions of patients using the drugs that affect renal function such as ACE inhibitors and ARBs were similar (Table 1), as well as other drugs (data not shown).

Moreover, the renal function of the patients in both the groups was significantly different. The mean serum creatinine was significantly lower in the patients with SGLT2i than in those with DPP4i (0.90 vs. 1.11 mg/dL; *p* value <0.001). Concordantly, the mean eGFR of the SGLT2i group was significantly higher than that of the DPP4i group (75.77 vs. 60.64 mL/min/1.73 m^2^; *p* value <0.001). Most patients in both the groups had eGFR levels ≥60 mL/min/1.73 m^2^; there were 299 patients (77.06%) in the SGLT2i group and 456 patients (65.99%) in the DPP4i group with eGFR ≥60 mL/min/1.73 m^2^. Other baseline characteristics were considered no difference between the two groups, including mean FBS, HbA1c, LDL cholesterol, HDL cholesterol, triglyceride, total cholesterol, AST, and ALT.

### 3.2. Renal Function

**Figure 1:**
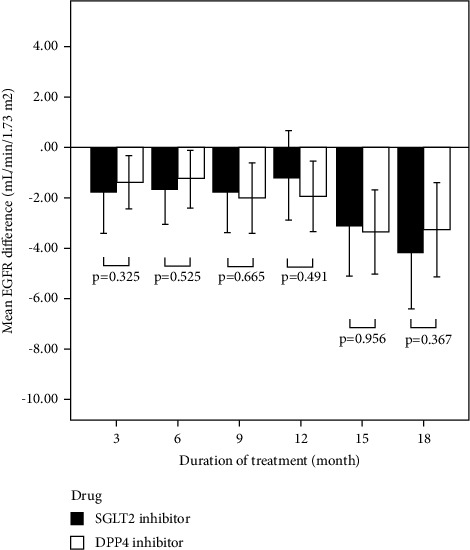
The mean differences in eGFR (mL/min/1.73 m^2^) from baseline at 3, 6, 9, 12, 15, and 18 months compared between patients using SGLT2i and DPP4i. The error bars represent a 95% confidence interval. A statistically significant difference was at *p*  <  0.05.

Table 2 indicates the mean eGFR differences between the two-time points throughout the study within the group, and Figure 1 presents the mean eGFR differences between the SGLT2i and DPP4i groups. Focusing on SGLT2i therapy (Table 2), the mean eGFR at the 3^rd^ month onward significantly dropped compared to the baseline, except at the 12^th^ month, with the magnitude of the decrease between 1.12 mL/min/1.73 m^2^ and 4.15 mL/min/1.73 m^2^. From the 3^rd^ month onward, there was no significant reduction in the mean eGFR, except for the comparison of the 12^th^ month compared to the 18^th^ month. Similarly, significant decreases in the mean eGFR from baseline were observed at every time point in patients using DPP4i (Table 3), with the magnitude of the decrease between 1.26 mL/min/1.73 m^2^ and 3.35 mL/min/1.73 m^2^. However, significant reductions in eGFR were observed in the 15^th^ and 18^th^ months compared to the 3^rd^ month. In addition, there were significant reduction between the 6^th^ and 15^th^, the 6^th^ and 18^th^, and the 12^th^ and 18^th^ months.

Although the results indicated a significant reduction in eGFR compared to baseline, the magnitude of the decrease was not significantly different between the patients using SGLT2i and DPP4i (Figure 1). For instance, the decreases in eGFR from baseline after 12 months of treatment with SGLT2i and DPP4i were 1.12 and 1.96 mL/min/1.73 m^2^, respectively (*p* value 0.491), and after 18 months, the decreases were 4.15 and 3.28 mL/min/1.73 m^2^, respectively (*p* value 0.367). However, at the 18^th^ month of treatment, there were 4.23% of the patients with a >25% decrease in eGFR in the SGLT2i group compared to 17.43% in the DPP4i group (Supplementary table 1).

**Figure 2:**
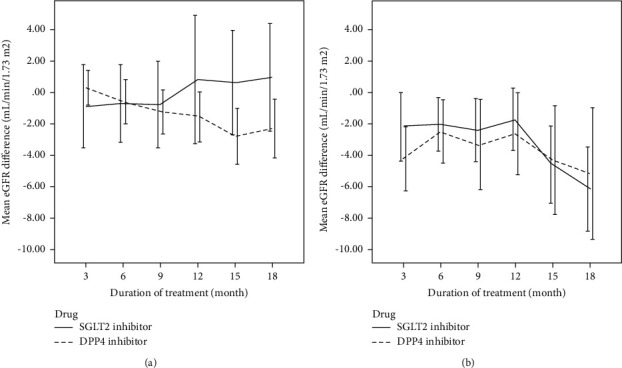
Subgroup analysis of the mean eGFR difference of the patients with baseline eGFR <60 mL/min/1.73 m^2^ (a) and ≥60 mL/min/1.73 m^2^ (b) who received SGLT2i and DPP4i for 3, 6, 9, 12, 15, and 18 months. The error bars represent a 95% confidence interval.

Subgroup analysis of the baseline eGFR indicated the difference in the trends of changes between the patients with baseline eGFR ≥60 mL/min/1.73 m^2^ and <60 mL/min/1.73 m^2^ (Figure 2). Focusing on the patients with baseline eGFR <60 mL/min/1.73 m^2^, the change in eGFR levels at the 18^th^ month of the patients using SGLT2i was greater than those using DPP4i (0.95 vs. −2.29 mL/min/1.73 m^2^, respectively, *p* value 0.125) despite no statistical significance. On the other hand, the patients with baseline eGFR ≥60 mL/min/1.73 m^2^ had less difference in eGFR changes between the SGLT2i and DPP4i groups (−6.16 vs. −5.20 mL/min/1.73 m^2^, respectively, *p* value 0.267).

### 3.3. Other Outcomes

**Figure 3:**
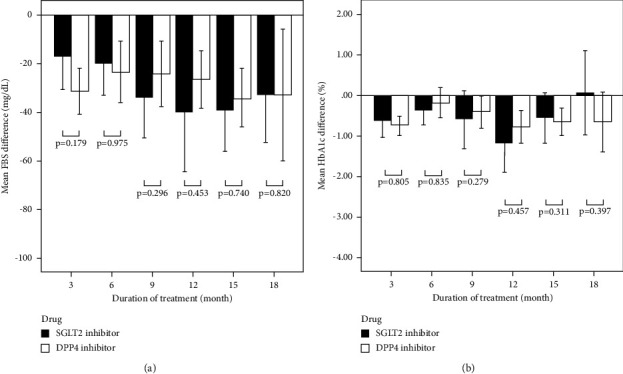
The mean differences in FBS (a) and HbA1c (b) levels from baseline at 3, 6, 9, 12, 15, and 18 months compared between patients using SGLT2i and DPP4i. The error bars represent a 95% confidence interval. A statistically significant difference was at *p*  <  0.05.

Figure 3 illustrates the differences in FBS (Figure 3(a)) and HbA1c (Figure 3(b)) levels in patients using SGLT2i and DPP4i at 3, 6, 9, 12, 15, and 18 months, compared to baseline. At the 18^th^ month of treatment, the average differences in FBS of the SGLT2i and DPP4i groups were −32.97 mg/dL and −33.16 mg/dL, respectively. Regarding HbA1c levels, patients using SGLT2i had an increase of 0.05%, while those using DPP4i had a decrease of 0.66%. However, both changes in the FBS and HbA1c levels were not significantly different between the SGLT2i and DPP4i groups in all visits.

## 4. Discussion

This real-world retrospective cohort study highlighted the significant declines in eGFR in T2DM patients who were treated with SGLT2is (i.e., dapagliflozin and empagliflozin) and DPP4is (i.e., vildagliptin and linagliptin) during the 18 months of follow-up. However, these eGFR reductions were not significantly different between the two drug groups. In addition, the reductions in eGFR observed in this study were less than 5 mL/min/1.73 m^2^ which unlikely contributed to detrimental effects to the patients.

Previous studies indicated that DPP4i could reduce the progression of albuminuria in patients with T2DM, but other renal benefits were similar to placebos, particularly the eGFR change [9, 10]. Moreover, DPP4is, such as vildagliptin and linagliptin, showed effects in cardiovascular risk reduction, especially in patients with high cardiovascular risk [17, 18]. Similarly, the current study showed significant reductions in eGFR from baseline at the end of the study, although it was not a rapid decline according to the Kidney Disease: Improving Global Outcomes (KDIGO) 2012 guideline [19]. The magnitude of eGFR reductions in this study was similar to that of the study by Lukashevich, who reported the eGFR declines between −1.62 and −1.98 mL/min/1.73 m^2^ in patients using vildagliptin for 12 months [20].

On the other hand, the renoprotective effects of SGLT2i have been reported in several randomised placebo-controlled studies [5, 6, 21–25]. Therefore, it was surprising that the patients in the SGLT2i group had a similar reduction in eGFR to the patients in the DPP4i group. In fact, a few studies reported controversial results comparing SGLT2i and DPP4i in terms of eGFR changes. For instance, the study by Esposito, et al. showed no difference in eGFR changes from baseline between the patients who used SGLT2i and DPP4i for 6 months [26]. It is possible that the renoprotective effect, i.e., the delayed reduction of eGFR, will be observed after 12–18 months of the treatment with SGLT2i according to some clinical trials [25, 27, 28].

The aforementioned studies indicated the same trends of eGFR changes among all SGLT2i therapies; a reduction in eGFR levels was shortly observed after initiating SGLT-2 inhibitors and reached the nadir within one to four months after initiation, followed by a return toward baseline. Then, the eGFR increased to a level comparable to the placebo after 12 to 18 months and remained stable or gradually declined compared to the placebo until the end of observation [25, 27, 28]. However, the eGFR levels of the patients using SGLT2i in the current study were not stable until the end of monitoring, similar to those with DPP4i. These eGFR declines were possibly due to the short time of data collection of the current study. Also, because this study was conducted in a real clinical setting, several factors, such as NSAID use and antihypertensive therapy, might confound the renal function of the patients using SGLT2i.

The subgroup analysis of the patients by their renal function suggested a potential factor in the renoprotective effects of SGLT2i. The patients with impaired baseline renal function, i.e., eGFR <60 mL/min/1.73 m^2^, who used SGLT2i seemed to gain more benefit than the patients with eGFR ≥60 mL/min/1.73 m^2^ in this study, even though the eGFR reduction was not significantly different from that in the DPP4i group. These results were in accordance with the KDIGO 2020 guideline, which advises the benefits of SGLT2i in T2DM patients with chronic kidney disease [2]. Moreover, the guideline recommends the continuation of SGLT2i in this group of patients, regardless of HbA1c levels.

Considering the glucose-lowering effects of the studied drugs, the results in this study indicated the reduction in FBS and HbA1c levels in patients using both SGLT2i and DPP4i compared to baseline. Moreover, the magnitude of the decrease in the FBS and HbA1c levels was not significantly different between the two drug groups. This study stated that both drug classes had antidiabetic effects and that the results suggested their potential renoprotective effect. Although the results showed a statistically significant reduction in eGFR, the average reduction was approximately 5 mL/min/1.73 m^2^. Nevertheless, it should be noted that the renal benefits of the drugs such as SGLT2i and DPP4i may partially depend on or are independent of their glucose-lowering effects [29, 30].

In contrast, several meta-analyses and reviews [15, 31, 32] of randomised controlled trials suggested the superiority of SGLT2i over DPP4i in renal outcomes, including the decreases in microalbuminuria, macroalbuminuria, worsening nephropathy, and progression of end-stage renal disease; however, those results were not statistically significant [31]. Although the abovementioned studies did not report the difference in eGFR levels as an outcome, there was a possibility that the two drug classes had an insignificant difference in eGFR preservation. In addition, the abovementioned meta-analyses did not include any study of vildagliptin [15, 31], so the results might be different from those of this study that included the patients using vildagliptin because vildagliptin treatment showed a significant decrease in albuminuria and an improved GFR after 24-week treatment [32].

In the era of SGLT2i, the results in this study suggested the possibility that SGLT2i might not be effective in eGFR preservation for all T2DM patients in real-world data. In addition, the adverse effects of SGLT2i, such as a urinary tract infection, should be considered when prescribing these drugs [33]. Due to the current results, SGLT2i should therefore be considered, especially in patients with impaired renal function, i.e., eGFR <60 mL/min/1.73 m^2^.

### 4.1. Strength and Limitation

This study is one of the studies that directly compared renal outcomes between SGLT2i and DPP4i, while most previous studies usually compared the drugs with a placebo. Furthermore, most previous studies usually focused on the rates of CKD diagnosis and 50% eGFR reductions, but this study emphasised the magnitude of eGFR declines. The study included the results of vildagliptin, while the renal effects of this drug are still lacking. Also, this is one of the few studies in real-world clinical settings in Thailand because both SGLT2i and DPP4i were not included in the National List of Essential Drugs of Thailand, meaning Thai patients who need to use the drugs have to pay themselves. Thereby, prescribing these drugs is mainly limited to tertiary and private hospitals.

Nonetheless, due to the retrospective methodology of this study, it possessed several limitations. First, some data could not be gathered and therefore were not included in the analysis, such as adherence of patients, duration of diabetes mellitus, and self-medication use. Consequently, there was a possibility of multiple confounding factors included in the analysis that could influence the results. On the other hand, these confounding factors could reflect the actual efficacy of the drug in real-world settings since it is impossible to control all factors in the real patient's life. Second, the difference in baseline characteristics, such as renal function and blood glucose, between the two groups should be taken into account. Because SGLT-2 inhibitors were available in Thailand much later than DPP-4 inhibitors, it is very difficult to find the same populations of patients. Finally, due to the availability of these drugs in the research setting, the duration of observation in this study is limited to only 18 months, so the results should not be extrapolated to long-term effects on eGFR. Longer duration of follow-up will be worth evaluating long-term effects on renal function, as well as a larger sample size. Also, additional patient data are needed to ameliorate confounding factors and prove the effects of SGLT-2 inhibitors on preserving renal function.

## 5. Conclusion

The findings of this study revealed the similarity in the reduction of eGFR between T2DM patients treated with SGLT-2 inhibitors and DPP-4 inhibitors for 18 months, as well as the reduction in FBS and HbA1c levels. However, patients with eGFR <60 mL/min/1.73 m^2^ who used SGLT-2 inhibitors demonstrated a smaller decline in eGFR than those who used DPP4i despite no statistical significance. Therefore, SGLT-2 inhibitors should be considered in patients with impaired renal function rather than in all T2DM patients.

## Figures and Tables

**Table 1:** Baseline characteristics of patients receiving SGLT2i and DPP4i.

Characteristics	SGLT-2 inhibitors (*n* = 388)	DPP-4 inhibitors (*n* = 691)
Male, *n* (%)	175 (45.10)	317 (45.88)
Age (year), median (IQR)	64 (49–79)	66 (50–82)
BMI (kg/m^2^), mean (95% CI)	26.70 (25.57–27.83)	25.76 (24.92–26.59)
Blood pressure (mmHg), mean (95% CI)		
Systolic	136.98 (133.91–140.05)	139.04 (136.16–141.91)
Diastolic	73.72 (71.82–75.61)	73.83 (71.82–75.61)
Never smoking, *n* (%)	294 (75.77)	451 (65.27)
Comorbidities, *n* (%)		
Hypertension	171 (50.59)	352 (50.94)
Dyslipidaemia	94 (24.23)	198 (28.25)
Coronary artery diseases	30 (8.88)	79 (11.43)
Concurrent medication, *n* (%)		
Metformin	121 (31.19)	206 (29.81)
Sulfonylureas	161 (41.49)	342 (49.49)
NSAIDs^*∗*^	217 (55.93)	291 (42.11)
ACEIs/ARBs	134 (34.54)	246 (35.60)
Renal function, mean (95% CI)		
Serum creatinine (mg/dL)	0.90 (0.84–0.96)	1.11 (1.02–1.20)
BUN (mg/dL)	15.66 (14.60–16.72)	17.76 (16.55–18.97)
eGFR (mL/min/1.73 m^2^)	75.77 (73.18–78.35)	60.64 (57.87–63.41)
FBS (mg/dL), mean (95% CI)	192.42 (180.76–204.08)	188.46 (176.17–200.75)
HbA1c (%), mean (95% CI)	8.82 (8.49–9.15)	8.50 (8.22–8.79)
Lipid profile (mg/dL), mean (95% CI)		
LDL cholesterol	124.36 (117.67–131.45)	118.86 (111.63–126.09)
HDL cholesterol	47.15 (45.24–49.06)	47.21 (44.69–49.74)
Triglyceride	159.76 (143.30–176.23)	166.36 (150.16–182.55)
Total cholesterol	190.19 (181.35–199.03)	184.59 (175.25–193.93)
Liver function (U/L), mean (95% CI)		
AST	26.45 (24.25–28.65)	26.77 (24.45–29.08)
ALT	24.97 (22.26–27.68)	25.46 (22.39–28.53)

^*∗*^NSAIDs include aspirin.

**Table 2:** Statistical analysis of the mean differences in eGFR (mL/min/1.73 m^2^) at each assessment time points, including baseline, and at 3, 6, 9, 12, 15, and 18 months after initiating SGLT2i.

Mean difference in eGFR	3 month (*p* value)	6 month (*p* value)	9 month (*p* value)	12 month (*p* value)	15 month (*p* value)	18 month (*p* value)
Baseline	−1.76 (0.006)	−1.66 (0.014)	−1.81 (0.008)	−1.12 (0.103)	−3.12 (0.004)	−4.15 (0.001)
3 month		−1.64 (0.105)	0.53 (0.663)	−2.09 (0.135)	0.01 (0.722)	−0.15 (0.689)
6 month			0.91 (0.885)	0.10 (0.894)	−0.95 (0.565)	−1.75 (0.196)
9 month				−0.62 (0.553)	−1.24 (0.281)	0.50 (0.925)
12 month					−0.61 (0.712)	−4.54 (0.025)
15 month						−1.89 (0.152)

Statistically significant difference at *p*  <  0.05.

**Table 3:** Statistical analysis of the mean differences in eGFR (mL/min/1.73 m^2^) at each assessment time points, including baseline, and at 3, 6, 9, 12, 15, and 18 months after initiating DPP4i.

Mean difference in eGFR	3 month (*p* value)	6 month (*p* value)	9 month (*p* value)	12 month (*p* value)	15 month (*p* value)	18 month (*p* value)
Baseline	−1.39 (0.010)	−1.26 (0.005)	−2.00 (0.001)	−1.96 (0.003)	−3.35 (<0.001)	−3.28 (0.001)
3 month		−0.55 (0.350)	−1.01 (0.124)	−1.19 (0.100)	−2.21 (0.010)	−3.38 (0.005)
6 month			−0.36 (0.189)	0.13 (0.551)	−1.88 (0.044)	−2.15 (0.013)
9 month				0.14 (0.823)	−0.49 (0.975)	−0.62 (0.384)
12 month					−1.63 (0.003)	−1.43 (0.152)
15 month						−0.39 (0.140)

Statistically significant difference at *p*  <  0.05.

## Data Availability

The data used to support the findings of this study are available from the corresponding author upon request.

## Supplementary Materials

Supplementary Materials Supplementary table 1: percentages of a decrease in eGFR at the end of treatment (18 months) between the patients receiving SGLT-2 inhibitors and DPP-4 inhibitors.
