# Supplementary material for: Renoprotective Effect of Thai Patients with Type 2 Diabetes Mellitus Treated with SGLT-2 Inhibitors versus DPP-4 Inhibitors: A Real-World Observational Study
Source: Adv Pharmacol Pharm Sci. 2023 May 15;2023:5581417. doi: 10.1155/2023/5581417 (PMC10202602; doi:10.1155/2023/5581417)
Supplement: Supplementary Materials — Supplementary table 1: percentages of a decrease in eGFR at the end of treatment (18 months) between the patients receiving SGLT-2 inhibitors and DPP-4 inhibitors. [file 5581417.f1.docx]

**Supplementary table 1** Percentages of decrease in eGFR at the end of treatment (18 months) between the patients receiving SGLT-2 inhibitors and DPP-4 inhibitors.

| **Percentage of eGFR decrease** | **SGLT-2 inhibitors (n, %)** | **DPP-4 inhibitors (n, %)** |
| --- | --- | --- |
| >25% | 17 (4.23) | 120 (17.43) |
| 25% to >10% | 98 (25.35) | 159 (22.94) |
| 10% to >0% | 158 (40.84) | 152 (22.02) |
| No decrease | 115 (29.58) | 260 (37.61) |
